# Neuroinflammation-Modulating Properties Combining Glutathione, N-Acetylcysteine, and Uridine Monophosphate in a Formulation Supplement: An In Vitro Study

**DOI:** 10.3390/brainsci15121340

**Published:** 2025-12-16

**Authors:** Simone Mulè, Francesca Parini, Rebecca Galla, Francesca Uberti

**Affiliations:** 1 Department for Sustainable Development and Ecological Transition, University of Piemonte Orientale, 13100 Vercelli, Italy; simone.mule@uniupo.it; 2 Noivita Srls, Spin Off, University of Piemonte Orientale, Strada Privata Curti 7, 28100 Novara, Italy; francescaparini00@gmail.com (F.P.); rebecca.galla@uniupo.it (R.G.)

**Keywords:** 3D in vitro models, food supplements, antioxidant effect, neuroprotective biomarkers, anti-neuroinflammation action, Transwell^®^ system, nervous health

## Abstract

**Background**: Neuropathic pain is a complex condition often resistant to current therapies due to limited efficacy and adverse effects. Nutraceuticals offer promising alternatives, combining antioxidant and anti-inflammatory properties with good tolerability. This study aimed to compare the effects of a commercial nutraceutical formulation, SUPERALA CARNITINE^®^ (Pharma Suisse Laboratories SpA, Milan, Italy), containing Alpha-Lipoic Acid (ALA), with a novel formulation, called SUPERALA CARNITINE^®^ Forte, where ALA and vitamin B6 were replaced by N-acetylcysteine (NAC), Glutathione (GSH), and Uridine monophosphate (UMP). **Methods**: An indirect gut–peripheral nerve axis was employed to simulate oral absorption, metabolism, and effect on nervous tissues using 3D in vitro models. Both formulations and their individual components were assessed for cytotoxicity and permeability in the gut model (Caco-2 cells in Transwell^®^) and, after gut metabolism, for antioxidant capacity, anti-inflammatory activity, and neuroprotective potential in the peripheral nerve model. **Results**: SUPERALA CARNITINE^®^ Forte improved cell viability and favoured the maintenance of intestinal integrity, showing enhanced permeability, and significantly reduced oxidative stress (OS) and pro-inflammatory cytokines (TNF-α, IL-2) at the peripheral nervous system. In addition, it increased levels of neuronal markers (p75, MPZ, NRG1, ERβ) and decreased NaV1.7 and NaV1.8 activity, indicating greater neuroprotection and analgesic modulation than the ALA-based formula. **Conclusions**: The replacement of ALA and vitamin B6 with NAC, GSH, and UMP produced favorable responses in vitro on neuronal cells, supporting a hypothetical potential interest in this nutraceutical combination and justifying further future in vivo investigations.

## 1. Introduction

Neuropathic pain is defined by the International Association for the Study of Pain (IASP) as “pain caused by an injury or disease of the somatosensory nervous system” [1,2]. This is a broad definition, encompassing more than 100 pathological conditions [2] and involving lesions spanning the entire neurological pain axis. Neuropathic pain is pain whose origin can be recognised as internal damage to the nervous system. The injury can occur at any level of the nervous system, including the peripheral nerves, spinal cord, and brain. About 30% of neuropathy cases occur because of diabetes. In addition, hundreds of other conditions, such as shingles, human immunodeficiency virus/acquired immune deficiency syndrome (HIV/AIDS), alcohol use disorder, and certain drugs, particularly chemotherapeutic agents used in cancer treatment, can also cause it. Recently, numerous molecular markers and experimental models have been identified that, with preclinical data, may improve the possibility of refining therapeutic strategies. Intracellular signalling, microglial activation, neurotrophic factors, increased levels of pro-inflammatory mediators such as cytokines, tumour necrosis factor α (TNF-α), interleukin-1β (IL-1β), chemokines, prostaglandins, and oxidative stress (OS) are among the mechanisms that explain the mechanisms underlying central neuropathy [3,4]. Although multiple experimental models are available to study inflammatory cascades, UV-induced inflammatory hyperalgesia is one of the models in which the involvement of key inflammatory mediators, including IL-1β, interleukin-2 (IL-2), interleukin-6 (IL-6), and TNFα, has been extensively characterized. Notably, these mediators are not restricted to the UV model, but represent common drivers of inflammation across in vivo pain models and clinical inflammatory conditions in both humans and rodents [5,6]. To improve the therapeutic strategy, experimental studies have also examined additional parameters. Pain treatment focuses on novel treatments and molecular pathways. While the cannabinoid system, through Gamma-Aminobutyric Acid (GABA)ergic modulation and cannabinoid 1 and 2 (CB1/CB2) receptors, plays a recognized role in regulating neuronal excitability and immune responses [7,8], recent evidence indicates that cannabinoid signaling may interact with purinergic pathways. This is particularly relevant in the context of bioactive elements such as uridine monophosphate (UMP), whose purinergic activity contributes to microglial modulation, cytokine regulation and neuronal repair. Therefore, the cannabinoid system provides a mechanistic background that complements the emerging role of UMP-mediated purinergic signaling in neuroinflammation and pain modulation [9,10]. In addition, the development of subtype-specific sodium channel blockers may yield a more successful therapeutic outcome. NaV1.7, due to its genetic links to pathological pain, and NaV1.8, because of its sensory neuron specificity, have been focused on as important in the pathophysiology of pain [11].

Neuropathic pain is still difficult to cure with current medications. Although there is evidence that medications with various mechanisms of action are effective in treating neuropathic pain, the extent of their effects is limited and frequently accompanied by side effects, making it difficult for many patients to receive pain relief at manageable doses [12]. Because of their low cost, strong nutritional and medicinal properties, and capacity to bind to a wide range of molecular targets, nutraceuticals, including dietary supplements and natural/herbal products, are therefore gaining popularity [13,14]. In this context, N-Acetyl-L-carnitine (N-ALC) is an endogenous compound with neurotrophic, neuroprotective, and metabolic roles. It is approved as a supplement or medicine depending on the country, mainly for peripheral neuropathies, due to its good safety profile. Beyond its antioxidant properties, N-ALC enhances oxidative balance, promotes acetylcholine synthesis, and supports nerve growth factor activity [15,16,17]. Animal studies show it aids nerve fiber repair, normalises Na^+^/K^+^-ATPase activity, improves nerve conduction, and prevents structural and age-related nerve damage [15,18]. Concurrently, comparable capability was observed following citicoline administration to enhance phosphatidylcholine production, which could promote the healing and repair of injured tissues [19,20].

In recent years, alpha-lipoic acid (ALA) has also become relevant in the context of the peripheral nervous system [21]. ALA is an enzymatic cofactor with powerful antioxidants and neuroprotective properties [22]. It is involved in cellular energy metabolism and has the unique ability to function in both aqueous and lipid environments, making it particularly effective at countering oxidative damage associated with many conditions, including neuropathic pain. ALA works on several fronts by promoting the neutralisation of these harmful molecules, protecting nerve cells, and, in addition, inhibiting pro-inflammatory cytokines, reducing neuroinflammation that contributes to pain perception [23,24]. Currently, ALA is under observation for several reasons, including its potential to influence glucose metabolism [25,26,27]. Although robust clinical trials and meta-analyses have not demonstrated a clinically relevant increase in hypoglycemic events [28,29], isolated post-marketing reports and rare immune-mediated reactions (such as insulin autoimmune syndrome, also referred to as Hirata syndrome) indicate that ALA may, in susceptible individuals, lower blood glucose levels more than expected [25,26,30]. This risk appears to be particularly relevant in patients receiving concomitant insulin or oral hypoglycemic agents and therefore warrants appropriate clinical caution despite the overall favorable safety profile observed in controlled studies [27,31]. In addition, it may also interfere with thyroid therapy and some chemotherapy. In some cases, it can cause gastrointestinal disorders (nausea, diarrhoea), skin rashes or dizziness [32,33]. For these reasons, pharmaceutical companies and scientific research have focused on the total or partial replacement of ALA with new substances that provide similar or improved modulation, prevention of neuropathic pain, and support of nerve function. Studies on antioxidants examined the protective, anti-inflammatory, and antioxidant properties of N-acetylcysteine (NAC) in the peripheral nervous system (PNS) [34]. NAC has beneficial effects that support essential cellular function across a variety of cellular systems. One of these benefits is increased intracellular glutathione (GSH) synthesis, which is known to be the body’s primary antioxidant [35]. Using integrated 3D intestinal–neuronal in vitro models, NAC and GSH were shown to cross the intestinal epithelial barrier and influence neuronal cells by modulating oxidative stress, inflammatory responses, and neuroprotective signaling pathways, including markers of myelination. These findings provide mechanistic evidence supporting a plausible causal link between intestinal uptake of these compounds and downstream neuronal effects, while acknowledging that direct confirmation in vivo is still required [34,35,36].

Based on the scientific evidence described above, our research focused on the pharmaceutical product SUPERALA CARNITINE^®^ (Pharma Suisse Laboratories SpA, Milan, Italy), available on the market, which contains ALA among its ingredients and is suitable for supporting nerve function. Specifically, this product was compared with a new oral formulation called SUPERALA CARNITINE^®^ Forte, in which ALA has been replaced with NAC, GSH, and UMP, a nucleotide associated with the inhibition of pain transmission in the spinal cord, exerting a marked antinociceptive effect in neuropathic pain models [37,38]. The research aimed to examine the ameliorative or maintenance effects of supplements following ALA replacement, using 3D models described and validated in the literature, by reconstructing in vitro all physiological phases that occur following oral intake to the target of interest, the nervous system, and analysing the neuroprotective and anti-inflammatory responses.

## 2. Materials and Methods

### 2.1. Agents’ Preparation

All samples from Pharma Suisse Laboratories SpA included two commercially available formulations, SUPERALA CARNITINE^®^ and SUPERALA CARNITINE^®^ Forte (referred to as SC and SCF in the results and discussion text and figures). These were compared both to each other and to individual agents. All test samples and human dosages are detailed in Table 1. Chemicals were dissolved in DMEM (Merck Life Science, Rome, Italy) without phenol red, supplemented with 2 mM L-glutamine and 1% penicillin–streptomycin (also from Merck Life Science, Rome, Italy). To mimic physiologically relevant exposure based on estimated systemic levels after oral intake, drugs were diluted 1:2000 for in vitro testing. This concentration was determined through preliminary range-finding experiments to avoid supraphysiological or cytotoxic effects [39,40,41]. For demyelination induction in 3D EngNT, 200 ng/mL of glial growth factor 2 (GGF, Tebu-Bio, Magenta, Milan, Italy) was added to the same medium [42].

### 2.2. Cell Cultures

Caco-2, human intestinal epithelial cells (ATCC, Manassas, VA, USA) were cultured in Adv DMEM-F12 (GIBCO^®^ ThermoFisher Scientific, Waltham, MA, USA) supplemented with 10% FBS, 2 mM L-glutamine, and 1% penicillin-streptomycin at 37 °C in a 5% CO_2_ incubator [43]. To maintain properties similar to intestinal absorption after oral intake, cells at passages 26–32 were used [44,45]. For each test, the cells were grown differently. 1 × 10^4^ cells were plated in 96-well plates for cell viability using an in vitro Toxicology Assay Kit based on MTT (Merck Life Science, Rome, Italy). Cells were cultured on a 6.5 mm Transwell^®^ insert with a 0.4 µm pore polycarbonate membrane (Corning Costar, New York, NY, USA) for absorption and integrity tests, measuring Trans-Epithelial Electrical Resistance (TEER).

RSC96, a rat-derived Schwann cell line (purchased from ATCC), was cultured in Adv DMEM (Thermo Fisher Scientific, Rodano, MI, Italy) containing 1% penicillin-streptomycin, 2 mM L-glutamine, and 5% FBS [46]. The culture was maintained at 37 °C with 5% CO_2_ and 95% humidity in a 37 °C incubator. Experiments utilised RSC96 cells from passages 10 to 15, which were sub-cultured two or three times weekly.

The ATCC rat neural PC12 cell line was cultured in Advanced RPMI-1640 medium (Thermo Fisher Scientific, Rodano, MI, Italy). This medium contained 5% FBS, 5% horse serum (HS; Merck Life Science, Rome, Italy), and 2 mM glutamine. Cultures were maintained at 37 °C in a 5% CO_2_ environment with 95% humidity, ensuring they remained below confluency. Assays utilised cells between passages 3 and 13 [47]. PC12 cells are commonly used for in vitro neuroprotective chemical screening [48], being a suitable neuronal cell line. To mimic a peripheral nerve environment, 4 × 10^6^ RSC96 cells were co-cultured with 1 × 10^5^ PC12 cells to create a 3D engineered neural tissue (EngNT) in vitro (see Section 2.6).

### 2.3. Experimental Protocol

**Figure 1:**
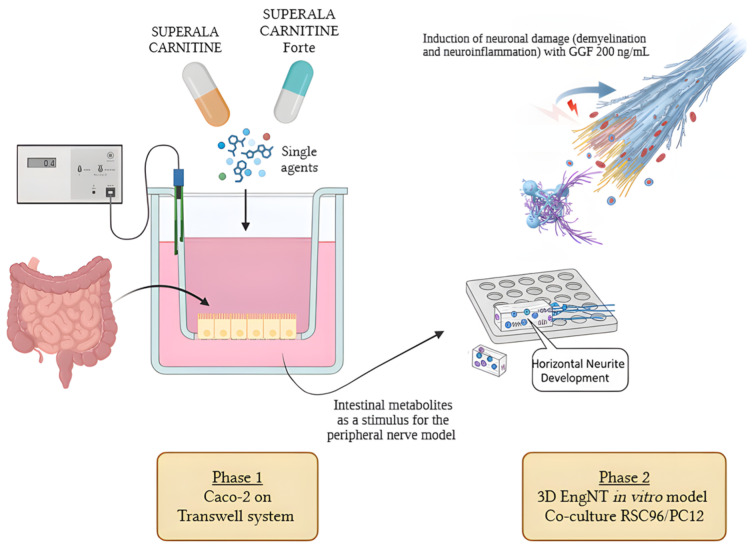
Schematic representation of the two analytical phases of the experimental protocol conducted in vitro.

The experiment had two phases, each with unique cellular compartments exposed to the test substances (Figure 1).

The investigation started with an in vitro intestinal barrier model using the FDA- and EMA-approved Transwell^®^ system to evaluate individual drugs and formulations [49,50]. To identify intestinal cytotoxicity, cell viability was measured with the MTT assay after 1–6 h of treatment. To check the integrity of the intestinal barrier, TEER must be measured accurately using the EVOM3^TM^ device. A fluorescent probe was employed to examine permeation across the barrier in various samples throughout this period. Additionally, metabolites from intestinal samples taken from the lower (basolateral) compartment of the Transwell^®^ system were collected, mimicking compounds in peripheral blood that either pass through the epithelium intact or are metabolised by the intestine. The physiological process of intestinal absorption was then modelled using this supernatant as a stimulus for the following cellular target, a 3D peripheral nerve co-culture with RSC96/PC12 cells (Phase 2).

In the second phase, the focus was on analysing the effects of the samples on the PNS. Starting on day 14 of maturation, GGF at 200 ng/mL was added to the EngNT 3D model to damage peripheral neurons and remove substantial myelin [51]. 3D EngNT was then treated for 24 h with the intestinal metabolite of each previously collected sample. The following analyses were carried out at this stage: cell viability by MTT test; OS by cytochrome C; protein markers associated with inflammation (TNFα and IL-2) by specific ELISA kits; peripheral neural neuroprotective biomarkers such as myelin protein zero (MPZ), p75, estrogen receptor β (Erβ) and neuregulin 1 (NRG) via specific ELISA kits and nociceptive and neuroinflammation related markers such as NaV1.7-NaV1.8 activities and GABA levels via specific ELISA kits.

### 2.4. In Vitro Intestinal Barrier Model

To assess whether drugs can cross the intestinal barrier in accordance with FDA and EMA regulations [49,50], a Transwell^®^-based intestinal barrier model was developed using a standard scientific procedure [43]. These methods are used to predict how many chemicals will be absorbed, metabolised, and bioavailable in humans after oral consumption. On both sides of the Transwell^®^ inserts, Caco-2 cells were maintained in complete medium that was changed every other day for 21 days prior to stimulation [51]. To assess mature intestinal epithelium and paracellular growth mechanisms, EVOM3^TM^ and STX2 electrodes (World Precision Instruments, Sarasota, FL, USA) measured TEER throughout. When TEER measurements exceeded 400 Ω · cm^2^ on the 21st day, absorption analysis was performed [52].

Before stimulation, the apical culture medium pH was 6.5, similar to the pH of the small intestinal lumen. Basolateral compartment (mimicking blood flow) was maintained at pH 7.4 [53].

Cells were stimulated with all substances for 1–6 h prior to analysis. A 0.04% fluorescent tracer (Santa Cruz, CA, USA) was used to assess permeability at each time point [54]. Caco-2 cells were incubated with the specified tracer concentration for 40 min at 37 °C to measure fluorescein transport. Fluorescence was then measured using a spectrophotometer (Infinite 200 Promplex, Tecan, Männedorf, Switzerland) with excitation at 490 nm and emission at 514 nm. Permeation rate was determined using this formula [54]:

J = Jmax [C]/(Kt + [C])

where

- Jmax: the maximum permeation rate;
- [C]: the initial concentration of fluorescein;
- Kt: the Michaelis–Menten constant.

Results are shown as mean ± SD (%), including negative controls without cells to eliminate Transwell^®^ membrane effects.

### 2.5. Cell Viability (MTT Test)

A standard method described in the literature [55] was employed to assess cell survival after each stimulation using the MTT-based In Vitro Toxicology Assay Kit (Merck Life Science, Rome, Italy). All solubilised materials, whether treated or untreated, were measured at 570 nm with a 690 nm correction using a spectrometer (Infinite 200 Pro MPlex, Tecan, Männedorf, Switzerland). Data were compared to the control. Results from five independent experiments performed in triplicate are presented as mean ± SD (%) of viable cells relative to the untreated control.

### 2.6. 3D EngNT In Vitro Model

In accordance with previous investigations, the 3D nerve tissue model was developed [46,56]. The relationships between RSC96 and PC12 cell lines are important for maintaining Schwann cells, promoting neurite regeneration, and replicating the peripheral nerve environment in vitro [56,57].

The scaffold was prepared using 80% Type I rat tail collagen (2 mg/mL in 0.6% acetic acid, Thermo Fisher, Milan, Italy), 10% MEM (Merck Life Science, Milan, Italy), 5.8% neutralising solution (Biosystems, Monza, Italy), and 4.2% Schwann cell suspension (4 × 10^6^ RSC96 cells per 1 mL gel). After the gel set, it was supplemented with 10% FBS, 100 U/mL Penicillin, and 100 µg/mL Streptomycin (Merck Life Science, Milan, Italy) in 10 mL DMEM (Merck Life Science, Rome, Italy) with phenol red. It was incubated at 37 °C with 5% CO_2_ for one day.

After the incubation time, plastic compression (120 g for 1 min) was used to stabilise the gel. After verifying the gel’s alignment and stability, it was divided into equal parts according to the samples needing handling. It was then placed into a 24-well plate once the Schwann gels had been aligned.

Each segment was plated with 1 × 10^5^ PC12 cells to form the co-cultures. Because it promotes horizontal neurite development, this portion is crucial. After neuronal cells were adhered to the collagen gel for 1 h at 37 °C, 1 mL of culture medium was added to each well of the 24-well plate containing the gels. Each segment was seeded with 1 × 10^5^ PC12 to create the co-cultures. But since it permits the neurites to develop horizontally, this stage is crucial. After neuronal cells attached to the collagen gel, one millilitre of culture medium was added to each well following a one-hour incubation at 37 °C.

### 2.7. ROS Production

In the EngNT in vitro model, superoxide anion release was assessed by measuring cytochrome C reduction [54]. Superoxide dismutase and cytochrome C (from Merck Life Science, Rome, Italy) were incubated for 30 min with both treated and untreated cells. Absorbance at 550 nm was recorded using a spectrometer (Infinite 200 Pro MPlex, Tecan, Männedorf, Switzerland). The results, averaged over five tests performed in triplicate, are expressed as the mean ± standard deviation (%) of lower cytochrome C nanomoles per microgram of protein relative to the control (0 line).

### 2.8. General Procedure for ELISA Assays

All ELISA analyses were performed according to the manufacturers’ instructions using specific commercial kits for each biomarker. Absorbance was measured at 450 nm using a microplate reader (Infinite 200 ProMPlex, Tecan, Männedorf, Switzerland). For each assay, a standard calibration curve (range depending on the specific kit) was used to determine analyte concentrations. For all ELISA kits, the results from five independent experiments, performed in triplicate, were expressed as mean ± SD (%) relative to the control (0 line). Only assay-specific details (kit type, matrix analyzed, standard range) are reported in the following paragraphs.

#### 2.8.1. TNFα Production ELISA Kit

TNF-α levels in 3D EngNT supernatants were quantified using the TNF-α ELISA Kit (Merck Life Science, Rome, Italy) [58]. The results were obtained using a calibration curve (range: 24.58–6000 pg/mL).

#### 2.8.2. IL-2 Production ELISA Kit

Following the manufacturer’s instructions, the Rat IL-2 ELISA Kit (FineTest, Wuhan, China) measured IL-2 levels in 3D EngNT lysates [59]. Colour intensity (OD) and standard concentration (31.25–2.000 pg/mL) were linked using standard curves.

#### 2.8.3. GABA ELISA Assay

GABA levels in 3D EngNT lysates were assessed using the GABA ELISA Kit (FineTest, Wuhan, China) [60]. Quantification was obtained through a standard curve ranging from 6 to 400 pg/mL.

#### 2.8.4. MPZ ELISA Assay

The MPZ content in 3D EngNT cell lysates was determined using the Rat ELISA kit (MyBiosource, San Diego, CA, USA) [51]. A standard curve with a range of 0.06 to 18 ng/mL was used to express the concentration as a percentage of ng/mL.

#### 2.8.5. p75 Expression by NGFR ELISA Assay

3D EngNT cell lysates were subjected to the Rat NGFR ELISA kit (MyBiosource, San Diego, CA, USA) in accordance with the product specifications [51]. Quantification was obtained through a standard curve ranging from 0.312 to 20 ng/mL.

#### 2.8.6. ERβ ELISA Assay

ERβ concentrations in 3D EngNT lysates were measured using the Rat ERβ ELISA Kit (Cloud-Clone, Houston, TX, USA) [61]. The result was given as % ng/mL against a 0.312–20 ng/mL standard curve.

#### 2.8.7. Neuregulin 1 (NRG1) ELISA Assay

NRG1 levels in 3D EngNT supernatants were measured using the FineTest NRG1 Rat ELISA Kit (Wuhan, China) [62]. Data were compared to a standard plot ranging from 0.156 to 10 ng/mL to obtain the results.

#### 2.8.8. NaV 1.7 ELISA Assay

NaV 1.7 was quantified on 3D EngNT lysates using the SCN9A/Nav1.7 ELISA Kit (LifeSpan BioSciences, Lynnwood, WA, USA) [63]. OD was correlated with the standards’ concentration (0.312 to 20 ng/mL) using a standard curve.

#### 2.8.9. NaV 1.8 ELISA Assay

On 3D EngNT lysates, the Mouse Sodium Channel Protein Type 10 Subunit Alpha (SCN10A) ELISA Kit (MyBiosource, San Diego, CA, USA) quantified NaV 1.8 [64]. Comparing colour intensity (OD) to standard concentration produced a standard curve.

### 2.9. Statistical Analysis

Data was presented as mean ± SD for at least five biological replicates per experimental method, with each replicate performed three times. The data were normalised to the control, averaging three replicates for each sample and dividing each value by the control mean. Results were expressed as a percentage-normalised value, with the control set to 0% to better interpret variations between treatment groups.

The mean optical density (OD) of each treated sample (three copies) was divided by the mean OD of the untreated control, which consisted of cells in culture medium and was used as the standard. Using the formula described above, the obtained ratios were multiplied by 100 and subtracted from 1 (the normalised control value) to obtain the percentage deviation from the control.

$$\%\;\mathrm{variation}\;=(\frac{\;\mathrm{mean}\;\mathrm{OD}\;\mathrm{treated}}{\;\mathrm{mean}\;\mathrm{OD}\;\mathrm{control}}-1)\times 100$$

The untreated control can be set to 0% to simplify normalization, and to analyse how treatments affect viability or metabolic activity across all datasets.

GraphPad Prism 10.2.3 used one-way ANOVA with Bonferroni’s post hoc, or Mann–Whitney U test, to compare groups. One-way ANOVA with Tukey’s post hoc test was employed to analyse TEER and viability data separately. A *p* < 0.05 was considered statistically significant.

## 3. Results

### 3.1. Evaluation of Formulations’ and Their Individual Components’ Biological Effects at the Level of a 3D In Vitro Intestinal Barrier Model

**Figure 2:**
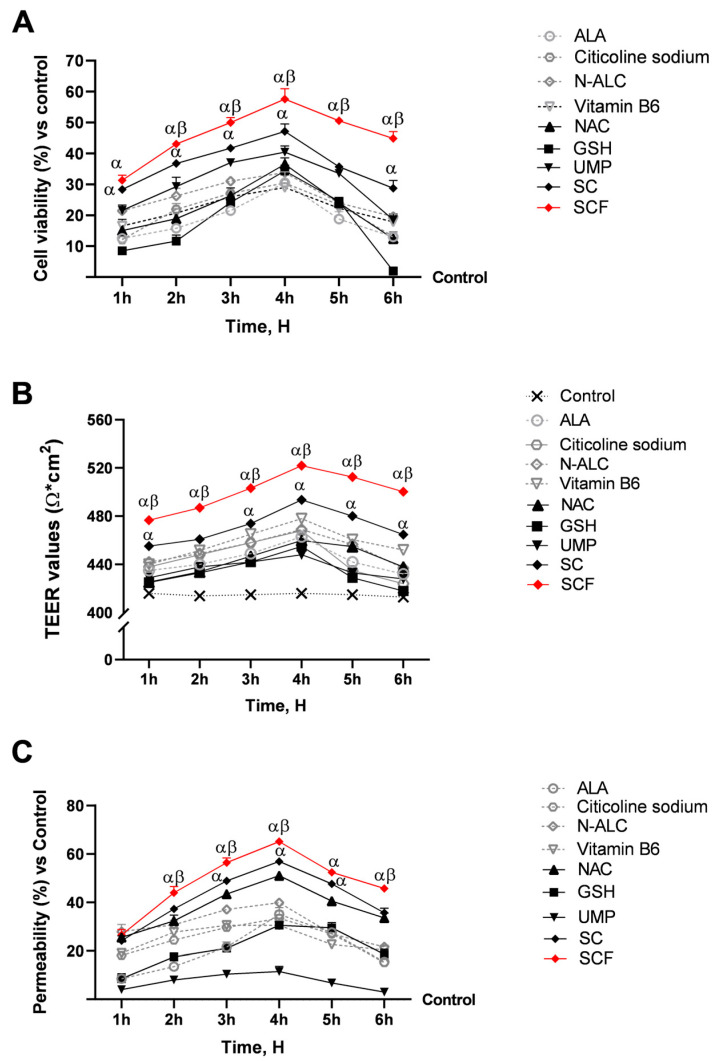
Outcomes following treatment within the 1–6 h interval for samples assessed using an in vitro 3D model of the intestinal barrier. In (**A**), the MTT assay measures cell viability; (**B**), the EVOM3™ device records TEER data; and (**C**), the fluorescent probe assesses permeability rate. Data are presented as mean ± SD (%) from 5 independent experiments in triplicate, normalised to the control (0% line only in (**A**,**C**)). α *p* < 0.05 vs. single agents; β *p* < 0.05 vs. SC.

Investigations were performed at the level of the intestinal compartment represented by a 3D Transwell^®^ model on which Caco-2 cells have been placed, in accordance with the experimental technique that was being considered for formulations designed for their oral use. This first step was crucial for understanding, and averting any cytotoxic effects of the test samples, as evidenced by altered cell viability and loss of intestinal epithelial integrity in the Transwell^®^ model. As shown in Figure 2, following treatment of the mature intestinal epithelium in the interval 1–6 h, all samples maintained cell viability, with a peak around 4 h of treatment. In detail, in Figure 2A, the best results were obtained on SCF, which recorded the most significant biological responses in terms of cell viability compared to all the individual agents and SC (*p* < 0.05). In detail, around the 4 h vitality peak, the SUPERALA CARNITINE^®^ Forte improved the biological responses by 47% compared to ALA, 30% compared to UMP, 37% compared to NAC, 40% compared to GSH, 41% compared to N-ALC, 50% compared to vitamin B6, and 18% compared to SC.

The EVOM3™ was used to obtain accurate TEER measurements of the intestinal epithelium during the 1–6 h treatment period (see Figure 2B). This was crucial for evaluating the integrity and function of the intestinal epithelial barrier. As shown in Figure 2B, all samples tested had TEER values exceeding 400 Ω·cm^2^ (*p* < 0.05), reflecting improved barrier integrity. Consistent with cell viability results from Figure 2A, data in Figure 2B reveal that SCF yielded the highest TEER, reaching 522 Ω·cm^2^ at 4 h (*p* < 0.05).

Figure 2C shows how SCF enhances each agent’s absorption kinetics (*p* < 0.05). After 4 h of treatment, all samples reached their peak absorption levels. NAC exhibited the highest permeability of all agents, reaching 51% at 4 h compared to the control (*p* < 0.05). SCF increased intestinal epithelial permeability around the peak of absorption by 46% in comparison to ALA, 82% in comparison to UMP, 22% in comparison to NAC, 53% in comparison to GSH, 40% in comparison to N-ALC, 53% in comparison to vitamin B6, and 13% in comparison to SC.

### 3.2. Analysis of the Biological Effects of Single Substances and Formulations at the Level of a 3D Model of the Peripheral Nerve

**Figure 3:**
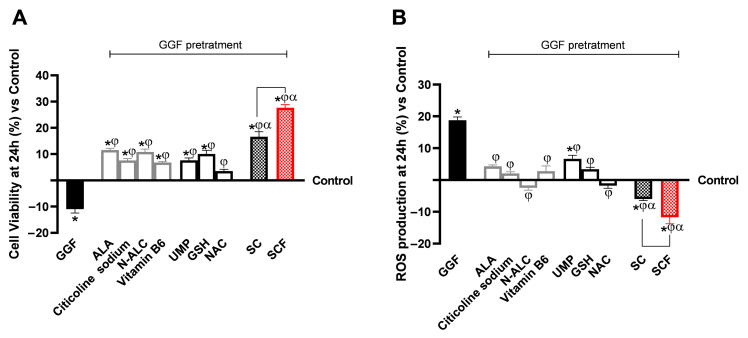
Cytotoxicity after 24 h test sample treatment in an in vitro 3D EngNT model under demyelination (GGF 200 ng/mL). (**A**) MTT assay cell viability at 24 h; (**B**) Cytochrome C-ROS production after 24 h. Results are shown as mean ± SD (%) from five triplicate experiments performed in triplicate, normalised to the control (0% line). * *p* < 0.05 vs. control; φ vs. GGF 200 ng/mL; α vs. single agents; the bar vs. SC.

The second group of analyses related to the experimental protocol focused on the effects of the samples on neuronal well-being, anti-inflammatory, and analgesic effects at the level of a 3D EngNT model of the peripheral nerve. 3D EngNT was initially pretreated with glial growth factor (GGF) at 200 ng/mL, which induced demyelination and initiated inflammatory and algesic pathways. Then, 3D EngNT was incubated with intestinal metabolites from each sample for 24 h, previously collected. First, following the treatment period, assessments of cell viability (Figure 3A) status and reactive oxygen species (ROS) production (Figure 3B) were conducted as performed in previous cell compartments. Pretreatment with GGF at 200 ng/mL decreased cell viability, resulting in elevated ROS levels compared to the control condition (*p* < 0.05). All individual agents were shown to counteract the neurotoxic effect induced by GGF at 200 ng/mL, improving cell viability (*p* < 0.05 vs. Control and GGF 200 ng/mL) and returning ROS levels closer to the control condition (*p* < 0.05 vs. 200 ng/mL). Regarding cell viability, among the individual agents examined as substitutes for ALA and vitamin B6, GSH showed an effect almost as strong as ALA and vitamin B6, with UMP showing effects even more similar. In the antioxidant response, GSH and NAC induced a more pronounced reduction, with NAC showing the greatest effect compared to ALA and vitamin B6 (*p* < 0.05 only for NAC vs. ALA and vitamin B6).

The formulations (SC and SCF) helped to implement the beneficial action of the individual components (*p* < 0.05), with better results from SCF than even SC (*p* < 0.05). In detail, SCF has promoted an improvement of cell viability compared to GGF 200 ng/mL and SC by 1.40-fold and 40%, respectively. In addition, regarding OS, it compared to GGF 200 ng/mL and SC contributed to reducing ROS production by 2.40-fold and 37%, respectively.

**Figure 4:**
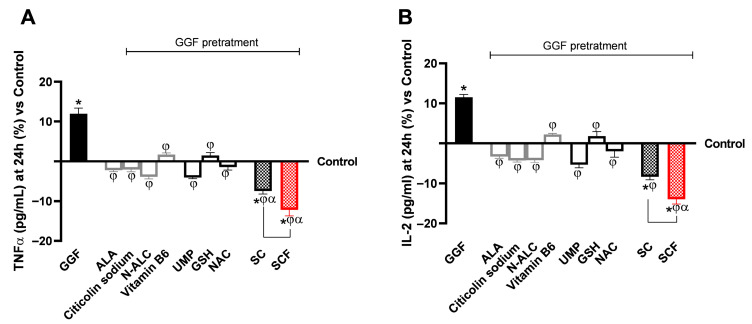
Neuroinflammation results after 24 h treatment with test samples in an in vitro 3D EngNT model under demyelination conditions (GGF 200 ng/mL). In (**A**,**B**), ELISA levels of TNFα and IL-2 were measured at 24 h, respectively. Data from five experiments, each performed in triplicate, are shown as mean ± SD (%), normalised to the control (0% line). * *p* < 0.05 vs. control; φ *p* < 0.05 vs. GGF 200 ng/mL; α *p* < 0.05 vs. single agents; and the bar *p* < 0.05 vs. SC.

As for the study on the anti-inflammatory effect of the test samples, their ability to modulate TNFα and IL-2 levels (pg/mL) was investigated (Figure 4A,B). Pretreatment with GGF at 200 ng/mL resulted in a marked increase in the levels of the two cytokines compared with the control condition (*p* < 0.05). The individual agents reduced the levels of the two measured proinflammatory cytokines, TNF-α and IL-2, compared with the GGF 200 ng/mL-induced neuroinflammation condition (*p* < 0.05). Better effects, consistent with the data on cell viability and ROS production, were observed after 24 h of treatment with both SUPERALA CARNITINE formulations, enhancing the protective action of the individual agents (*p* < 0.05). Regarding TNFα production, among the individual ALA and vitamin B6 replacement agents, UMP showed the most pronounced reduction, superior to ALA, followed by NAC with an effect slightly inferior to ALA. Comparable effects were also observed in terms of anti-neuroinflammatory effect on IL-2 levels.

SCF, in which ALA was replaced with NAC, GSH and UMP, was shown to elevate the anti-inflammatory effect of SC (*p* < 0.05). For TNFα, SCF promoted an increase in the reduction in cytokine levels by 39% compared with SC and 2-fold compared with GGF 200 ng/mL. At the same time, SCF promoted an increase in the reduction in IL-2 levels, also by 40% compared with SC and 2-fold compared with GGF 200 ng/mL.

**Figure 5:**
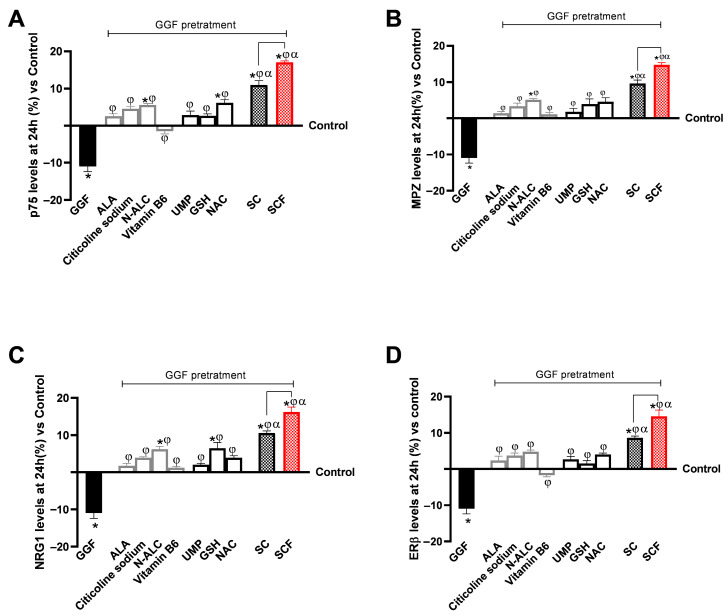
24 h neuroprotection biomarker results from in vitro 3D EngNT demyelination (GGF 200 ng/mL) test samples. p75 levels in (**A**), MPZ levels in (**B**), NRG1 levels in (**C**), and ERβ levels in (**D**) were all measured using ELISA kits at 24 h. The data, normalised to the control (0% line), are shown as mean ± SD (%) from five independent tests conducted in triplicate. * *p* < 0.05 vs. control; φ *p* < 0.05 vs. GGF 200 ng/mL; α *p* < 0.05 vs. single agents; the bar *p* < 0.05 vs. SC.

Further analysis was conducted using the 3D EngNT model to gain deeper insight into the neuroprotective responses following 24 h of treatment with the test samples. In this part, using specific ELISA kits, the levels of markers such as p75, MPZ, NRG1, and Erβ were measured (Figure 5A–D). Consistent with previously obtained data, pretreatment with GGF resulted in a marked reduction in the levels of all examined biological markers of neuronal well-being. For all markers, GGF levels at 200 ng/mL were reduced by about 10% compared to the control condition (*p* < 0.05). As evidenced by Figure 5A–D, all individual agents examined demonstrated an active role in modulating survival, neuronal well-being and plasticity consistent with previously obtained data on the inflammatory context. All the individual agents that replace ALA and vitamin B6 cause an increase in p75 signal that is comparable to ALA (UMP and GSH; *p* < 0.05 vs. vitamin B6) or even greater, such as NAC (*p* < 0.05 vs. ALA and vitamin B6). Analysis of MPZ also revealed similar effects (Figure 5B), with NAC and GSH having a stronger, more noticeable impact than ALA and vitamin B6. While GSH had more biological effects than ALA and vitamin B6 alone for NRG1 (Figure 5C,D), NAC, GSH, and UMP exhibited results that were comparable to ALA and better than vitamin B6 (*p* < 0.05) for Erβ levels.

As previously observed, both SC and SCF formulations enhanced the effect of individual agents with a sharper gap compared to GGF 200 ng/mL (*p* < 0.05). In all analyses conducted, SCF obtained the most significant results compared with the respective SC (*p* < 0.05). SCF promoted an average increase in levels of about 40% over their respective ALA-based Formula for all study parameters; in particular, in Figure 5B, MPZ levels, SCF improved by about 45% over the SC, which represents the largest relative increase observed within this set of measurements.

**Figure 6:**
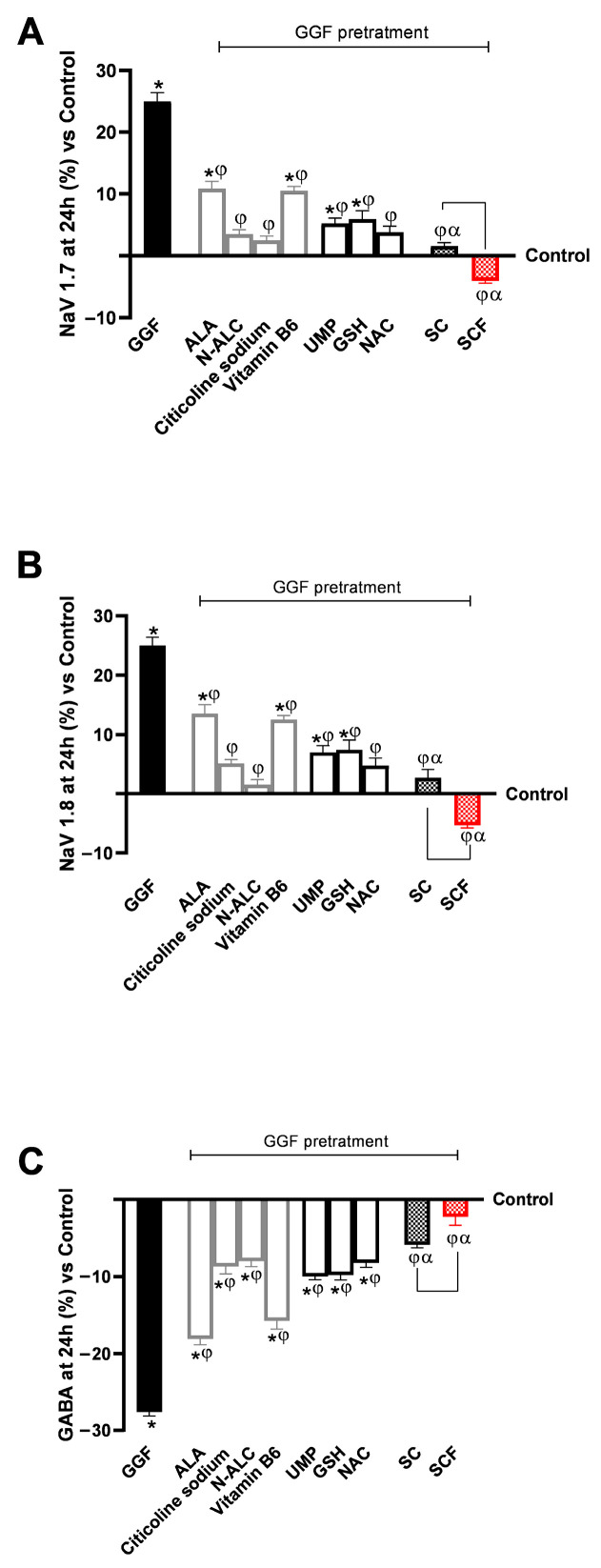
Effects on nociception-related biomarkers after a 24 h treatment with test samples on an in vitro 3D EngNT under demyelination conditions (GGF 200 ng/mL). (**A**–**C**) show 24 h ELISA measurements of NaV 1.7, NaV 1.8, and GABA, respectively. Data are presented as mean ± SD (%) from five triplicates, normalised to the control (0% line). * *p* < 0.05 vs. control; φ *p* < 0.05 vs. GGF 200 ng/mL; α *p* < 0.05 vs. single agents (excluding SC vs. N-ALC for NaV 1.8 activity); the bar *p* < 0.05 vs. SC.

Finally, as the last part of the analysis at the level of the 3D EngNT model pretreated with GGF, crucial research was conducted on parameters directly related to the manifestation of nociception and neuronal damage, inflammation. In this context, the analysis of NaV 1.7 and NaV 1.8 activity in association with GABA presence following 24 h of treatment was crucial. As can be seen from Figure 6, the pre-treatment with GGF 200 ng/mL has helped towards a favourable molecular context for the induction of the nociception pathway, clearly inhibiting the levels of GABA (*p* < 0.05), while contributing to a clear increase in the levels and activity respectively of NaV 1.7 and NaV 1.8 (*p* < 0.05). All individual agents played an active role in counteracting the pro-nociceptive effect induced by GGF at 200 ng/mL (*p* < 0.05). All replacement samples (NAC, GSH, and UMP) with ALA and vitamin B6 demonstrated neuroprotective and hypothetically anti-nociceptive effects at the peripheral nerve level compared to ALA and vitamin B6 alone (*p* < 0.05).

For all the markers examined, the two formulations of SC allowed to improve the effects of individual agents by reducing the negative and neurotoxic effect promoted by GGF 200 ng/mL (*p* < 0.05), in terms of reduction in the levels and activity, respectively, of NaV 1.7 and NaV 1.8 and increase in the presence of GABA in this case to values close to the control condition. After 24 h of stimulation with SCF, the best results were obtained compared to SC (*p* < 0.05). In detail for both NaV 1.7 and NaV 1.8, SCF reduced, respectively, their levels and activity by 50% compared to SC and by 4 times compared to GGF 200 ng/mL. At the same time, it increased GABA levels by 60% compared to SC and 13-fold compared to GGF 200 ng/mL.

## 4. Discussion

The current medications for neuropathic pain management and neuronal function support have limited efficacy and significant negative effects. Because plant derivatives and pharmaceutical compounds have anti-inflammatory, antioxidant, and neuroprotective qualities, research has investigated combination therapy with these molecules [65]. Nutraceuticals are natural products used as nutritional supplements in the dietary supplement industry. Since they are frequently concentrated or refined substances that are administered as pills, tablets, or other pharmaceutical forms and may offer certain health benefits, the term “nutraceuticals” is derived from the terms “nutrition” and “pharmaceutical” [66].

Analysing the safety status of the various agents and formulations was the first step when considering an oral supplement, such as SC and SCF. The bioactive components of interest and oral formulations were initially metabolised by reproducing the intestinal absorption pathway before evaluating the effects on the target nervous compartments. Nowadays, evaluating novel formulations or compounds with limited oral bioavailability can lead to indirect safety and reproducibility concerns. Limited absorption often necessitates compensatory higher dosing or alternative administration routes, which can increase experimental variability; inter- and intra-individual differences in systemic exposure, material usage, and the potential for off-target effects, without implying a direct mechanistic link between bioavailability and toxicity [67,68]. Therefore, it is necessary to understand and predict the complex biological processes that occur from the mouth cavity to the gut and within the cellular compartments of interest to correctly conceive and develop new oral drugs or supplements. Animal testing remains the gold standard in preclinical research because it can mimic the absorption, distribution, metabolism, and excretion (ADME) processes mediated by systemic blood circulation [69]. Although in silico models can estimate certain molecular properties relevant for absorption and permeability, it remains challenging to account for the multitude of factors that influence a molecule’s behavior across complex biological barriers in vivo. To address these limitations, 3D in vitro cell culture systems have been developed to better replicate native tissue function, offering complementary and more predictive platforms for drug testing [70,71].

In the intestinal context, the study used a 3D in vitro Transwell^®^ model with intestinal Caco-2 cells, which allows substances to be studied, sorted, and classified under controlled conditions, according to the biopharmaceutical classification system, and to obtain significant absorption and safety data at the human intestinal level [72]. Through this experimental model, it was possible to demonstrate that for both groups of test samples, both individual agents and both test formulations of SC and SCF did not induce any cytotoxic effects in the intestine. All formulations studied enhanced maintenance of intestinal function and integrity, as assessed by TEER, a widely used parameter for assessing tight junction (TJ) function. Indeed, TEER values provide an estimate of the resistance to ionic flow through the epithelial layer, yielding a measure of barrier function relative to tissue structure and thickness [73]. The TEER measurement demonstrates increased permeability through mature epithelium, with no damage or alteration of the biological barrier, with the formation of holes that could compromise and alter absorption data. Through the study, it was possible to determine that, in the 1–6 h range, SC and SCF enhanced the transepithelial permeability of their individual components, reaching a peak at 4 h. This effect likely reflects synergistic interactions within the multi-component formulation, including improved solubility, microenvironmental stabilization, reduced efflux, and increased apparent permeability, consistent with observations in both Caco-2 monolayers and 3D intestinal models of combined nutraceuticals [74,75,76]. A slight decrease in barrier function was observed at 6 h; a transient pattern likely reflects the temporal dynamics of compound action rather than any cytotoxic effect. Several mechanisms may explain this phenomenon. First, the rapid absorption and metabolism of bioactive ingredients, such as ALA, NAC, GSH and others, can lead to a maximal transient effect on intestinal and endothelial barrier function at around 4 h, followed by a modest decline as intracellular levels of active metabolites decrease [72,77,78,79,80]. Cells may initially respond to compound stimulation with transient increases in metabolic activity, tight junction protein expression, and antioxidant defenses (e.g., GSH), enhancing TEER and barrier integrity. Subsequent reductions likely reflect homeostatic feedback, physiological adaptation. Additionally, partial degradation or metabolism of the compounds in the culture medium may slightly modulate barrier function over time [79,81,82]. For all the parameters examined, already at the intestinal level the substitution of ALA and vitamin B6 with NAC, GSH, and UMP in SCF allowed for to improvement of safety and potency of the formulation, assuming a possible combined effect among bioactive ingredients (already visible in SC), improved and confirming what has been achieved at this level for NAC-GSH [34,36].

The safety and cytotoxicity data obtained at the intestinal level, without loss of bioactive potential due to cell metabolism, allowed continuation at the nervous level (PNS) in an in vitro 3D model (3D EngNT), seeking improvements in the treatment of induced neuroinflammation (GGF 200 ng/mL) and nociception. 3D EngNT is a hydrogel used in nerve healing to deliver Schwann cells or therapeutic Schwann cell-like cells. It also holds promise as a model for pre-clinical in vitro screening [83,84]. When neurons and 3D EngNT are co-cultured, neurite growth is encouraged and direct, mimicking essential characteristics of the peripheral nerve environment distal to a peripheral nerve repair [57]. This strategy could serve as a model for drug screening by standardising a scalable co-culture process and creating reliable protocols [46]. Indeed, previous studies have shown the remarkable applicability of this co-culture model both to known drugs such as Ibuprofen, with dose–response patterns similar to those observed in other in vitro studies [46,85], and to the nutraceutical context, where biological effects are investigated by pretreating the co-culture with GGF at 200 ng/mL [62,86,87]. As earlier investigations have shown, the favourable contrasting action of natural extracts at the level of the 3D EngNT, where it was initiated by myelinization, has validated the possible use of GGF 200 ng/mL in disease modelling [42]. By offering a drug-screening platform for the development of novel treatments, as suggested by the literature, the model validates its substantial applications in motor neuron disorders. SCF has helped to maintain the health of the nerve model under conditions of damage and demyelination, allowing for the amplification of the effect of SC. In detail, by reducing the levels of TNFα, IL-2, improving the production and presence at the nervous level of peripheral nerve function biomarkers such as p75, MPZ, NRG1 and Erβ. This allows the survival of damaged neurons, neuronal plasticity, Schwann cell proliferation, and maintenance of myelination [88,89]. The results observed can be attributed to NAC’s function. NAC is well-known for its ability to counteract acetaminophen, but less is known about how it can lessen neuropathic pain and neuroinflammation [90]. Regarding this biological aspect, TNFα and IL-2 serve as robust readouts of peripheral nerve activity, reflecting endogenous neuronal and Schwann cell responses to oxidative or metabolic stress. Their production captures key neuroinflammatory and paracrine signalling processes, which are critical for neuronal survival, axonal regeneration, and glial–neuronal crosstalk, thus providing physiologically meaningful insights into the impact of bioactive compounds such as NAC and GSH [91,92,93]. One of the primary targets of NAC in the nociceptive pathway occurs via indirect regulation of nuclear factor kappa B (NF-κB) [35]. Activation of NF-κB during inflammation ultimately triggers the transcription of inflammatory genes such as TNFα and IL-1β [94]. By reducing ROS, IKappa kinase (IKK) complex and enhancing intracellular glutathione, NAC attenuates NF-κB activation, thereby limiting the transcription of pro-inflammatory genes such as TNFα and IL-1β. This effect is context-dependent and may vary with cell type, stimulus, and NAC concentration [95,96]. Because NAC prevents NF-kB activation, it suppresses the production of cytokines, including TNFα, IL-1β, and IL-2, which are important for the inflammatory pathway and the development of peripheral neuropathy [97]. It should be mentioned that NAC’s interaction with N-ALC, an ingredient in the original formulation (SC), is responsible for the enhanced impact of SCF after ALA and vitamin B6 were substituted with NAC. Due to their antioxidant, neuroprotective, and glutamatergic transmission-modulating properties, NAC and N-ALC have been found to have a potential synergistic mechanism for neuropathic pain [90,98]. Research both in vitro and in vivo shows that while NAC attenuates oxidative stress and neuroinflammation in models of nerve damage and diabetic neuropathy [99,100], N-ALC promotes axonal regeneration and the expression of mGlu2 receptors, reducing nociceptive sensitivity [101]. Due to these peculiarities, the combination of NAC and N-ALC has been evaluated in preclinical models, showing a synergistic effect in reducing oxidative stress, glial activation and neuronal degeneration, resulting in improved cell survival and axonal regeneration compared to individual treatments [102].

In addition to the above, NAC has been shown to reduce the negative effects of ROS by serving as a precursor to GSH, an antioxidant known for its potent anti-inflammatory and antioxidant properties [103]. These bioactive effects discussed and observed in the study contributed not only to an improvement of the cellular environment by reducing fading and oxidative stress but also to the levels of biomarkers of peripheral nerve health; indeed, inflammation and oxidative stress lead to an imbalance of MPZ, ERβ, and NRG1 contributing to peripheral damage, neuroinflammation and pain. MPZ is a key structural protein of the myelin sheath, which helps to conduct nerve impulses and insulates nerve fibers [104]. Increased nociception sensitivity and compromised nerve function can result from myelin breakdown caused by oxidative stress and inflammation. The growth and development of nerves are among the cell signalling pathways in which these receptors are engaged. Neuropathy and impaired nerve regeneration can result from disruption of ERβ signalling [105]. In the formation and upkeep of myelin and nerve function, NRG1 is essential [106]. NRG1 signalling imbalances can cause nerve injury and worsen pain [107].

Regarding the pathways correlated with neuroinflammation and nociception, the bioactive effects through the reduction in NaV 1.7, NaV 1.8 activity and increase in GABA levels for SC and SCF after intestinal metabolization were maintained. As observed in other studies on neuropathic pain, voltage-gated sodium channels NaV1.7 and NaV1.8, expressed in dorsal root ganglia neurons, show increased activity and a reduced activation threshold, contributing to neuronal hyperexcitability and spontaneous generation of action potentials [108]. This hyperactivity is associated with a reduction in GABAergic inhibitory tone; however, in vitro studies have shown that increased GABA availability or activation of GABA A/B receptors reduces spontaneous discharge and normalises the function of NaV1.7/1.8 channels [109]. In our 3D EngNT peripheral nerve model, GABA may also act on Schwann cells expressing functional GABA receptors, modulating paracrine signaling, myelin protein expression, and neuroinflammatory responses. This includes indirect engagement of GABAergic pathways via GABA A receptors, normally expressed in peripheral nerves [110]. These effects, by bioactive ingredients, highlight that changes in GABA levels not only reflect neuronal activity but also capture the integrated functionality of the neuron–glia network, providing a physiologically meaningful reading of neuroprotective or analgesic interventions [111,112,113]. This is in line with what was observed in vitro in our conducted study. In this scenario, UMP-based Formula (SCF) shows possible anti-nociceptive action, successfully addressing damage to the peripheral myelin sheath. Animal studies demonstrate that UMP and cytidine improve neuromuscular recovery and modulate spinal nociceptive transmission, reducing pain perception. These effects are thought to involve P2Y nucleotide receptor agonism (e.g., UDP), which can attenuate neuronal excitability and nociceptive signaling in the spinal cord. In models of neuropathic pain, nucleotide receptor agonists significantly reduce pain perception. In neuropathy models, extracellular uridine and Schwann cell nucleotide receptors interact to activate the molecular machinery that alters glial cells’ cytoskeleton [37,114]. These results suggest a synergistic effect of SCF components, including NAC and GSH, consistent with both our previous work and independent studies, demonstrating their combined antioxidant and neuroprotective actions in neuronal and peripheral nerve models [34,36,115,116].

The bioactive effects of SC and SCF formulations demonstrated beneficial combination actions provided by the newly added components without any toxicity or loss of efficacy due to intestinal metabolism. The experimental approach was validated when the 3D EngNT model yielded optimal results. Concurrently, additional in vivo or clinical research will be required to validate the bioactive effects of the new formula compared with the existing formula. As in vitro models lack the complexity of the human body, including interactions with immune, endocrine, and nervous systems, these experiments provide mechanistic insights but require confirmation in more physiologically relevant systems.

## 5. Conclusions

The study conducted highlighted the potential of the new formulation SUPERALA CARNITINE^®^ Forte as a viable alternative to the current product containing ALA and vitamin B6 (SUPERALA CARNITINE^®^). Analysis of biological parameters in in vitro models showed that replacing ALA and vitamin B6 with NAC, GSH, and other bioactives improved cell viability and intestinal permeability. After intestinal metabolization, SUPERALA CARNITINE^®^ Forte showed a greater antioxidant and anti-inflammatory effect on neuronal targets than SUPERALA CARNITINE^®^, helping to reduce OS and the production of pro-inflammatory cytokines, such as TNF-α and IL-2. Furthermore, results in nerve cells suggest that these formulations may modulate neuropathic pain mechanisms by engaging key receptors, such as sodium channels NaV1.7 and NaV1.8. These data indicate that the combination approach of NAC, GSH, UMP, and other bioactives may be a promising strategy for supporting nerve function and managing neuropathic pain, with superior effects compared with ALA and vitamin B6 combined in a food supplement (SUPERALA CARNITINE^®^). However, further preclinical and clinical studies will be needed to confirm the efficacy and safety of SUPERALA CARNITINE^®^ Forte in more complex human-related conditions.

## Figures and Tables

**Table 1:** Composition of the supplement formulations under investigation.

SUPERALA CARNITINE^®^ (SC)			SUPERALA CARNITINE^®^ Forte (SCF)		
Sample	Dosage	In Vitro	Sample	Dosage	In Vitro
N-Acetyl L-Carnitine (N-ALC) of which:	1204 mg	602 µg/mL	N-Acetyl L-Carnitine (N-ALC) of which:	1204 mg	602 µg/mL
L-Acetyl-Carnitine	1000 mg	500 µg/mL	L-Acetyl-Carnitine	1000 mg	500 µg/mL
Citicoline sodium (oral grade; granular) of which:	284 mg	142 µg/mL	Citicoline sodium (oral grade; granular) of which:	284 mg	142 µg/mL
Citicoline	250 mg	125 µg/mL	Citicoline	250 mg	125 µg/mL
Vitamin B6 HCL of which:	3.44 mg	1.72 µg/mL	5′-UMP disodium SALT-Freeman of which:	77 mg	38.50 µg/mL
Vitamin B6	2.80 mg	1.40 µg/mL	UMP	50 mg	25 µg/mL
ALA 30-60 Mesh	800 mg	400 µg/mL	NAC USP	600 mg	300 µg/mL
			GSH	200 mg	100 µg/mL
Total (Bioactive ingredients)	2052.80 mg		Total (Bioactive ingredients)	2100 mg	

## Data Availability

The data presented in this study are available on request from the corresponding author (The Laboratory of Physiology carefully stores raw data to ensure permanent retention under a secure system).

## Abbreviations

The following abbreviations are used in this manuscript:

Adv DMEM-F12	Advanced Dulbecco’s Modified Eagle’s Medium/Nutrient F-12 Ham’s
Adv RPMI	Advanced Roswell Park Memorial Institute medium
Adv DMEM	Advanced Dulbecco’s Modified Eagle’s Medium
ALA	Alpha-Lipoic Acid
ATCC	American Type Culture Collection
CB2R	Cannabinoid Receptor 2
DMEM	Dulbecco’s Modified Eagle’s Medium
DMEM-F12	Dulbecco’s Modified Eagle’s Medium/Nutrient F-12 Ham’s
EMA	European Medicines Agency
EngNT	Engineered Neural Tissue
ERβ	Estrogen Receptor β
FBS	Foetal Bovine Serum
FDA	Food and Drug Administration
GABA	Gamma-Aminobutyric Acid
GGF	Glial Growth Factor
GSH	Glutathione
IASP	International Association for the Study of Pain
IKK	IKappa kinase
IL-1β	Interleukin-1β
IL-2	Interleukin 2
MPZ	Myelin Protein Zero
MTT	3-(4,5-Dimethylthiazol-2-yl)-2,5-Diphenyltetrazolium Bromide
N-ALC	N-Acetyl L-Carnitine
NAC	N-Acetylcysteine
NF-κB	Nuclear factor kappa B
NRG1	Neuregulin 1
OS	Oxidative Stress
PNS	Peripheral Nervous System
ROS	Reactive Oxygen Species
SC	SUPERALA CARNITINE^®^
SCF	SUPERALA CARNITINE^®^ Forte
TEER	Trans-Epithelial Electrical Resistance
TJ	Tight Junction
TNFα	Tumour Necrosis Factor α
UMP	Uridine monophosphate
